# Determining the impact of 24/7 phone support on hospital readmissions after aortic valve replacement surgery (the AVRre study): study protocol for a randomised controlled trial

**DOI:** 10.1186/s13063-017-1971-y

**Published:** 2017-05-30

**Authors:** Irene Lie, Stein Ove Danielsen, Theis Tønnessen, Svein Solheim, Marit Leegaard, Leiv Sandvik, Torbjørn Wisløff, Jonny Vangen, Tor Henning Røsstad, Philip Moons

**Affiliations:** 1 Centre for Patient-centered Heart and Lung research, Department of Cardiothoracic Surgery, Division of Cardiovascular and Pulmonary Diseases, Oslo University Hospital, Building 63, Ullevål, Oslo, Pb 4956, Nydalen 0424 Norway; 2 Institute of Clinical Medicine, Faculty of Medicine, University of Oslo, Oslo, Norway; 3 KU Leuven Department of Public Health and Primary Care, KU Leuven – University of Leuven, Leuven, Belgium; 4 Department of Cardiothoracic Surgery, Division of Cardiovascular and Pulmonary Diseases, Oslo University Hospital, Ullevål, Oslo, Norway; 5 Department of Cardiology, Division of Medicine, Oslo University Hospital, Ullevål, Oslo, Norway; 6 Department of Nursing and Health Promotion, Faculty of Health Sciences, Oslo and Akershus University College of Applied Sciences, Oslo, Norway; 7 Oslo Centre for Biostatistics and Epidemiology (OCBE), Oslo University Hospital, Oslo, Norway; 8 Department of Infectious Disease Epidemiology and Modelling, Norwegian Institute of Public Health, Oslo, Norway; 9 Department of Health Management and Health Economics, University of Oslo, Oslo, Norway; 10 Institute of Health and Care Sciences, University of Gothenburg, Gothenburg, Sweden

**Keywords:** Thoracic surgery, Patient readmission, Clinical trial

## Abstract

**Background:**

Patients undergoing surgical aortic valve replacement (sAVR) have high rates of 30-day readmissions. They also report a low health-related quality of life (HRQOL) and elevated anxiety and depression. The aim of the AVRre study is to determine the efficacy and cost of a 24/7 phone-support intervention in reducing post-discharge readmissions after sAVR. The nature of the support is to help patients better understand and self-manage non-urgent symptoms at home.

**Methods/design:**

AVRre is a prospective, randomised controlled study comprising 30 days of continuous phone-support intervention and then intermittent follow-up for the first 12 months. Phone call data from and to patients are evaluated qualitatively; thus, the study has a mixed-method design. Two hundred and eighty-six patients, aged >18 years, scheduled for a sAVR — singly or in combination with another procedure — are recruited from locations in southeast Norway. Patients are randomly assigned to the intervention group, who are purposively phone-called individually 2 and 9 days after discharge and offered on-demand 24/7 (around-the-clock) telephone support for 30 days post-discharge. The primary outcome variable is the number of 30-day hospital readmissions. Secondary outcomes are anxiety and depression symptoms, as measured by the Hospital Anxiety and Depression Scale, HRQOL and quality-adjusted life years, measured by the EuroQol (EQ-5D). Intervention and hospital readmission (diagnosis-related groups (DRGs)/length of stay) for the first year after initial discharge from hospital are used for a cost-utility analysis. Standard parametric and non-parametric tests are used for evaluations over time. Analysis of covariance is used to control for possible differences at baseline. Narratives from phone calls are transcribed verbatim and analysed using systematic text condensation.

**Discussion:**

A complex ‘around-the-clock’ intervention within a university hospital-based setting could be an effective strategy to reduce the high readmission rates to hospital after sAVR. Furthermore, the AVRre 24/7 phone-support manual can be adapted to other high-risk surgery populations with high readmission rates.

**Trial registration:**

ClinicalTrials.gov, NCT02522663. Registered on 11 August 2015.

**Electronic supplementary material:**

The online version of this article (doi:10.1186/s13063-017-1971-y) contains supplementary material, which is available to authorized users.

## Background

Severe aortic stenosis that demands surgical aortic valve replacement (sAVR) due to considerable morbidity and mortality is increasing in prevalence as the elderly population increases globally [1]. sAVR remains the definitive treatment for aortic stenosis (AS), and sAVR has an estimated annual incidence of 85,000 cases [1] in the USA and 1500 cases in Norway (unpublished data from Norwegian Heart Surgery). Irrespective of good immediate surgical outcomes, sAVR patients are characterised by high rates of 30-day readmissions to hospital after discharge. For example, the rates are 19.6% in a US population [2] and 26% in a Danish population [3], and from unpublished register data in Norway (Norwegian Patient Registry, AVR patients’ readmission to hospital, 2011–2014, the Norwegian Directorate of Health 2016), it is estimated to be 22.4% in Norway. Reasons for 30-day readmissions after sAVR are available in two studies. In an American study, heart failure, cardiac rhythm disorders, stroke or transient ischaemic attack, pneumonia, pneumothorax/pleural effusion and gastrointestinal bleeding were reported [4]. In a Danish study, atrial fibrillation, pericardial effusion, congestive heart failure and pneumonia were the most dominant reasons for 30-day readmissions; these conditions occurred acutely in 25% of cases [3]. One in five patients in a study after major surgery was readmitted to a non-index hospital. The use of an index hospital with specialised competence, versus non-index re-hospitalisation, resulted in significantly lower in-hospital mortality [5].

Readmission to hospital in Norway is defined as an unplanned, emergency admittance 8 hours to 30 days after discharge from hospital, accompanied by at least one overnight stay with the readmittance [6]. The majority of patients (96%) discharged approximately 1 week after complex sAVR return home intending to be responsible for their own physical and mental health and for arranging follow-up by their general practitioner (GP) when needed. However, following discharge, patients/inhabitants and partners experience insecurity and the psychological and physical burdens associated with potential readmissions. Moreover, the estimated cost of readmissions is 2 billion Norwegian kroner (NOK) each year, with an estimated readmission rate of approximately 20% [6].

The clinical experience of specialists and municipal healthcare services reveals that standard care at discharge does not typically include patient education. Two systematic reviews and meta-analyses of randomised controlled trials (RCTs) that sought to reduce 30-day hospital readmissions for different diseases concluded that no single intervention (e.g. education, telephone follow-up) was associated with reduced risk for 30-day re-hospitalisation [7, 8]. For example, Melton et al. (2012) suggested a two-time telephone follow-up after discharge during office time [9]. More complex, high-methodological quality interventions, ones in which patients are educated and receive support for self-care, are recommended for preventing hospital readmission and increasing health-related quality of life (HRQOL) status, which otherwise is poorly self-reported [3].

Research on readmission after heart surgery highlights a great need for interventions to be implemented during the first 30 days after discharge to ensure that patients receive quality healthcare and engage in safe practices [3, 5, 10–13]. In a Norwegian home-based intervention the first month after cardiac surgery (*n* = 185), patients and relatives pointed to several negative factors, including lack of information at discharge, insecurity and lack of a ‘connection’ to the index hospital. This was especially true in the first month after surgery, if complications such as pleural effusion and arrhythmias appeared post-discharge [14].

Furthermore, the Norwegian patient experience surveys (2016) report that almost 50% of patients received incomplete information related to discharge preparation, especially regarding what symptoms to expect after discharge, and how and whom to contact if complications occur [15]. These experiences may contribute to feelings of anxiety in patients. Indeed, approximately 29–61% of all patients experience moderate to severe levels of anxiety and depression during the first month after cardiac surgery, with symptoms remaining elevated up to 6 months following surgery [16, 17]. These factors deserve our attention, because anxiety and depression are predictors of morbidity and mortality after heart surgery [18–21]. Therefore, one can hypothesised that interventions that target patients’ and relatives’ need for information and follow-up during the first month after cardiac surgery and the provision of these interventions around the clock could avoid unnecessary hospital readmissions. Indeed, a 24/7 follow-up service by phone goes beyond the results of regular telephone follow-up during office time. No study has tested the effect of an around-the-clock follow-up intervention, where the patients’ needs and symptoms are the base for the intervention. Expert healthcare professionals will be able to assess worsening of symptoms on the phone before a critical stage, and patients can be advised to contact a GP. Telephone follow-up also allows for inclusion of patients who live a long distance from both index and non-index hospitals.

This paper presents the detailed protocol for the AVRre study, in which we aim to determine the efficacy and cost utility of 30-day around-the-clock, 24/7 phone-support intervention after discharge for sAVR. The study’s design and protocol are in accordance with the current Standard Protocol Items: Recommendations for Interventional Trials (SPIRIT) guidelines [22]. A SPIRIT checklist is available online for this manuscript (see Additional file 1). Results will be reported following the CONsolidated Standards Of Reporting Trials (CONSORT) guidelines for non-pharmacological interventions [23, 24].

## Study objectives

### Primary objective

The primary objective of this study is to determine whether a 30-day, around-the-clock, 24/7 phone-support intervention reduces the number of hospital readmissions 30 days after discharge from hospital. The intervention begins immediately after initial discharge, and the outcomes of patients in the intervention are compared to a control group, which receives usual care.

### Secondary objectives

The secondary objectives of the study are as follows:

- To determine whether an around-the-clock, 24/7 phone-support intervention implemented within 30 days after discharge reduces objectively measured symptoms of anxiety and depression compared to a control group in the first year after discharge from hospital
- To determine whether the around-the-clock, 24/7 phone-support intervention implemented within 30 days after discharge improves HRQOL and quality-adjusted life years (QALYs) compared to the control group in the first year after discharge from hospital
- To perform an economic evaluation specifically to (1) determine the cost utility of the intervention compared to usual care in the study population and (2) assess the cost of readmission to hospital and the cost of GP consultations during the first year after discharge for the intervention and the control groups

## Methods/design

### Study design

AVRre is a prospective, randomised controlled trial (RCT), comprising 30 days of intervention and 12 months of follow-up. The main study began in August 2015. As the intervention consists of phone calls from patients to hospital and vice versa, the design of the study includes an explorative, qualitative component. Thus, this study employs a mixed-method study design. Supporting material for the AVRre study is provided in Additional file 2.

### Study population, recruitment, randomisation and follow-up

Patients eligible for study participation are 18 years or older and are referred for sAVR surgery for the first time at Oslo University Hospital, at either the Ullevål or Rikshospitalet locations, the largest hospitals in southeast Norway. Consecutively admitted sAVR patients are asked by project nurses to participate, and they are included if they meet the following criteria: (1) the surgery is an elective treatment with a single AVR (biological (b) or mechanical (m), an AVR (b or m) + aortocoronary bypass or an AVR (b or m) + supracoronary tube graft; (2) the patient can understand, speak and write the Norwegian language and (3) can be contacted by phone after discharge from hospital. Exclusion criteria are the following: (1) the patient has been admitted to an intensive care unit (ICU) for more than 24 hours; and/or (2) has complications related to surgery (e.g. surgery caused cerebral insult with significant impact on cognitive functions).

One to three days before the planned sAVR, patients arrive at hospital for preoperative preparations. During this time, the project nurse informs the patients about the aim and process of the study. The patients are then given the informed consent form and the baseline questionnaires for review and are given time to consider participation in the study. Patients are contacted a second time before surgery to answer any questions about the study and to deliver further information about the study.

After the patient has provided written informed consent, patient assignment to either usual care (control) or intervention is accomplished by a web-based randomisation system developed and administered by the Unit of Applied Clinical Research, Institute of Cancer Research and Molecular Medicine, Norwegian University of Science and Technology, Trondheim, Norway. This system has been approved by the Data Protection Officer at Oslo University Hospital as complying with human experimental subject protections. Randomisation (1:1 ratio) is performed consecutively with block randomisation and varying size of the blocks to make it impossible to predict to which group the patients are likely to be allocated. Randomisation is done without stratification to the two cardiothoracic sites of the study hospital (i.e. Ullevål or Rikshospitalet).

**Fig. 1:**
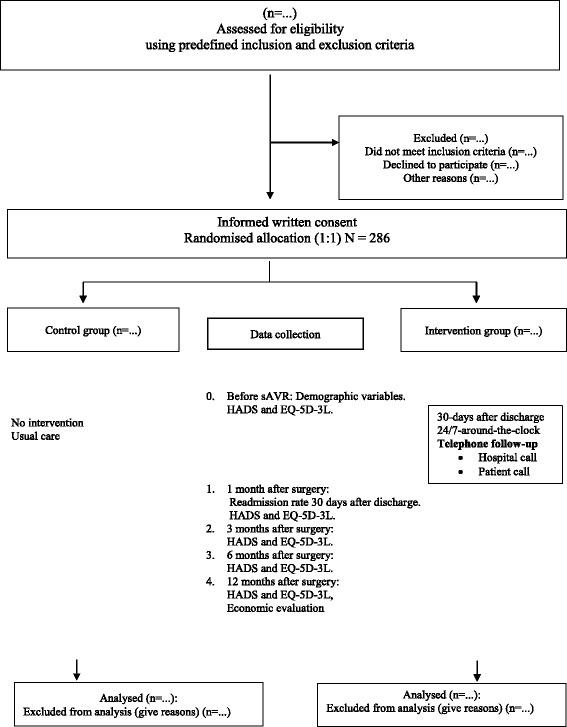
Patient flow and data collection chart

Before standard discharge from the university hospital to the patient’s local hospital on the fourth day post-sAVR, the project coordinator (SOD) informs the patient verbally and in writing (with a leaflet) to which group he/she has been allocated. For both the control and the intervention groups, the follow-up assessment takes place 1 month (T1), 3 months (T2), 6 months (T3) and 12 months (T4) after discharge from hospital. Follow-up consists of mailing by postal questionnaires with prepaid stamps for return post after completion (see the patient flow and data collection chart of Fig. 1).

### Usual care

Preoperatively, all patients recommended by a thoracic surgeon to have aortic valve replacement surgery receive information on expected HRQOL improvement, longer life expectancy and possible complications of surgery. Currently in Norway, at discharge, there is no standard information or post-discharge telephone support in usual care, not from nurses, doctors, university hospitals or local hospitals. There is no blinding in the study.

### Intervention: two components

A brief description of the development of the 24/7 phone-support manual and an example section of it is available online (Additional file 1). The intervention consists of two components.

#### Component 1

Patients in the intervention group are purposely called on days 2 and 9 after discharge by the project coordinator to proactively assess the patient’s present condition and to determine if the patient has questions or if problems have emerged. Details in the patient’s medical history are compiled in advance and reviewed prior to the phone calls, and each structured call relays reminder information to the patient about the availability of the 24/7 phone-call service as part of the intervention. The project coordinator emphasises the importance of daily physical activity [3] and how it has a positive effect on rehabilitation, morbidity and mortality after sAVR. When the patient receives a Short Message Service (SMS) one day ahead of the phone call, he/she has the opportunity to respond if the scheduled day or time is inconvenient for them. Data from the phone calls will be collected in a written, standard format. Also, patients will be encouraged to relate their individual responses/experiences/narratives, for example, when they experience anxiety symptoms.

#### Component 2

The intervention group is also offered 24/7 around-the-clock telephone availability during the entire first month post-discharge. Volunteer, expert intensive care nurses on duty in a cardiac ICU have been trained by an interdisciplinary team to answer the calls from sAVR patients during the first month after discharge. One aspect that the ICU nurses make extremely clear is that this 30-day 24/7 phone-support provision is not a replacement for emergency calls to 113 (911 in some countries). For ethical reasons and because of hospital responsibility to the patients, we include some ’red’ responses (i.e. acute or emergency) in the manual. However, we expect that the sAVR patients will call the intervention phone line mostly for non-urgent health information. If a patient’s problems demand advanced expertise, the project nurse will consult the thoracic surgeon or cardiologist on call in hospital to ensure that accurate diagnostics are completed and suggestions for treatment are made. The project coordinator is always available for the ICU nurses to consult, and will take initiative to arrange regular follow-up meetings and interdisciplinary discussion of challenging phone calls.

### Variables, sources and measurement

**Fig. 2:**
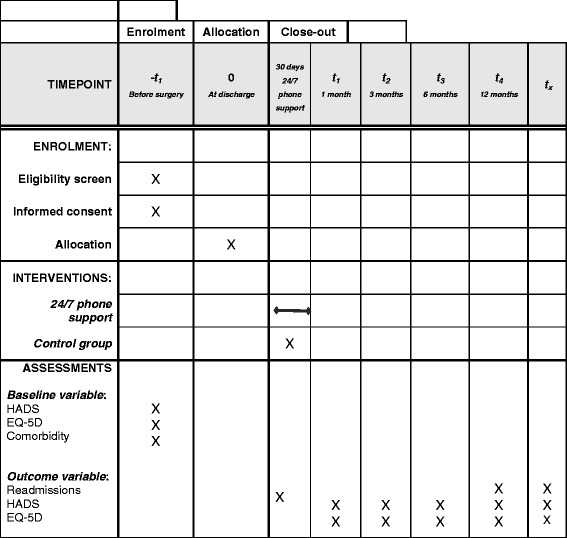
SPIRIT schedule of enrolment, interventions and assessments for the AVRre study

Patients will be longitudinally assessed five times during the course of the project: before sAVR and 1, 3, 6 and 12 months after discharge for sAVR. Additional file 1 includes a SPIRIT checklist for the schedule of enrolment, intervention and assessments as presented in Fig. 2. The written informed consent form for the AVRre study is included as Additional file 3.

### Primary outcome

#### Readmission

Data from the Norwegian Patient Registry (NPR), the Norwegian Directorate of Health and patients’ medical records will be used to gather the numbers of readmissions within 30 days after sAVR discharge. Moreover, data on causes of readmissions (ICD-10 codes), time and location (index and non-index hospital) will be collected.

### Secondary outcomes

Anxiety and depression symptoms are measured using the Hospital Anxiety and Depression Scale (HADS) [25, 26], a standardised, self-report instrument consisting of 14 items in two subscales. The 14 items include seven items for anxiety (HADS-A) assessment and seven for depression (HADS-D). Patients rate themselves on each item from 0 (not present) to 3 (maximum), yielding a total possible score of 21 for each subscale. The psychometric properties of the HADS are well documented in research conducted in many different countries; this includes valid use in heart patients [26].

HRQOL and QALY are assessed using the internationally recognised EQ-5D instrument [27]. EQ-5D is a standardised instrument comprising five dimensions of self-reported health status for clinical and economic appraisal. These dimensions are mobility, self-care, usual activities, pain/discomfort and anxiety/depression. The respondent rates himself on each dimension for the degree (no problem, some problem, extreme problem) that best describes his/her present health status.

#### Economic evaluation

Economic analyses are performed for two reasons. (1) The time it takes to proactively call the patients, as well as the time needed to answer the patient on the intervention phone and the time needed for calling back if consulting the physician at hospital, will be measured and valued. (2) Register data on the cost of readmission to hospital (diagnosis-related groups (DRGs)/length of stay) and the number of GP consultations during 30 days and the first year after discharge will be used for the cost-utility analysis and will be reported as an incremental cost-effectiveness ratio (ICER). Sensitivity analyses will be conducted to measure uncertainty in the estimates. In addition, data from the patients’ medical records are gathered, e.g. comorbidities.

### Data management and statistical analysis

The first and second author have the daily responsibility for overseeing patient safety, study design, database integrity and study conduct and have access to the final study dataset. No data will be entered before the intervention is finalised, to make sure that the baseline data will not influence the intervention. A random check of at least 20% of entered data will be performed to ensure data quality before starting the full data analysis. Data are presented as means ± standard deviations for continuous variables and percentages for nominal variables. The primary outcome variable is measured using the chi-square test to evaluate group differences. The secondary outcome variables are measured longitudinally to assess changes over time. Symptoms of anxiety and depression (HADS) will be analysed in continuous-form variables before being transformed to a cut-off score ≥8 for anxiety and depression respectively. We will apply a multilevel logistic model with the time nested within the patient, and Hosmer’s step-down procedure [28] to establish a final model. Analyses will be conducted in R version 3.3.2 (2016-05-03, R Core Team, 2016) (https://www.r-project.org/). Mixed model analyses will be applied for repeated measurement of anxiety (yes/no) or depression (yes/no) using HADS and EQ-5D [29]. Analysis of covariance (ANCOVA) is used to test mean changes between groups, controlling for possible differences at baseline [30, 31]. A paired sampled *t* test is used to analyse mean changes within groups. A statistic will be considered significant when the corresponding *P* value is <0.05. The Statistical Package for Social Sciences (SPSS), version 21 (released 2012, IBM Corp., Armonk, NY, USA) is used for statistical analysis.

### Missing data

The amount of missing data in the study and the methods used to handle missing data in the analysis will be reported [32]. Complete registry data will be available on primary outcome readmission 30 days after discharge for sAVR. If a patient dies within 30 days, it will be counted as readmission. Out of a total sample of 286 sAVR patients, we estimate 0–2 deaths. These numbers will not influence the power of the study. Regarding secondary outcomes, the guidelines in the article of Little et al. [32] will be followed; hence, we will perform multiple imputation analyses in analyses where missing data are not handled properly otherwise. In addition, sensitivity analyses will be performed to assess the robustness of assumptions made.

### Narrative data analysis

#### Data/narratives from patients’ phone calls to hospital and project coordinator phone calls to patients

All qualitative data are transcribed verbatim and analysed in several steps using systematic text condensation in accordance with the approach of Malterud [33]. Experts in qualitative analysis in the research group responsible for the AVRre study will re-read the narratives independently before the subsequent data reduction into meaning units, condensed meaning units, subthemes and themes guided by the study’s aim.

### Mixed methods

Qualitative data as narratives from the patients are intended to complement and enrich the quantitative data from study measures. Using narratives from patients’ phone calls will focus on the spontaneous needs and symptoms from the patients’ perspective, thus avoiding recall bias that may occur during interviews at a later time. The two approaches are planned to be used in tandem to answer the research questions in this study [34]. One challenge that needs to be figured out is how to interpret conflicting results.

### Sample size and power calculation

In 2013, a total of 503 patients had aortic valve replacement surgery at Oslo University Hospital. To estimate the sample size required to make confident conclusions about the primary outcome — the number of readmissions 30 days after discharge from hospital — we used published data on readmissions in Norway for patients >65 years old. Seventeen percent of the patients are readmitted to hospital within 30 days from discharge [6]. A sample size of 286 patients with 143 patients in each group will achieve at least 80% power to detect an expected difference of 15% in the control group and 5% in the intervention group at the 5% significance level using the chi-square test.

### Ethical considerations: ethics and disseminations

The study is conducted in accordance with the Declaration of Helsinki. Ethical approval was obtained from the Regional Committees for Medical and Health Research Ethics (approval 2013/2031-3). All patients receive both verbal and written information about the aim of the study and are informed that they are free to withdraw from the study at any time. Patients sign an informed consent document prior to inclusion. The codebook with study numbers and person-sensitive information and data from phone calls is kept in a locked, firewall-secured cabinet. To be able to perform the cost-utility analysis, we included in the written, informed consent form the patients’ permission to collect person-identifiable sensitive data from the medical record and from the Patient Registry Department at the Norwegian Directorate of Health. The results are presented so that the identity of the subjects cannot be identified, either directly or from derived information. Results from this study will be published in peer-reviewed journals.

## Discussion

This randomised controlled study, which we call AVRre, is the first programme to offer and test the effectiveness of a complex 24/7, around-the-clock intervention to optimise sAVR patients’ safety and healthcare in the vulnerable readmission phase 30 days after discharge. The intervention is complex, because phone calls are made proactively to the patient 2 and 9 days after discharge, and because telephone support from expert healthcare professionals is made available day, evening and night during the first 30 days after hospital discharge. Combining experimental and explorative approaches results in mixed-method data, which will strengthen the conclusions we can draw and produce more solid information about sAVR patients’ experiences at home.

Analyses of patient narratives about the symptoms they experience and their needs during early rehabilitation will produce new insights for developing effective patient information systems and education programmes relevant for sAVR patients in the future. Moreover, symptom monitoring combined with evidence-based and clinical expertise advice can accommodate patients’ desires to feel secure and to submit their requests for information after discharge from hospital [14]. Moreover, as we have hypothesised, this should reduce the number of 30-day hospital readmissions and reduce symptoms of anxiety and depression. Readmissions after sAVR are sparsely documented in the research literature, and reports of readmissions in RCTs, except for a few registry studies/observational studies, are almost unknown [8].

Mixing both quantitative and qualitative methods in this RCT increases the probability of obtaining valuable empirical knowledge from sAVR patients in addition to evidence of treatment effect [34]. First, triangulation generated by different data sources is possible; e.g. suppose a patient in the intervention group has a high score for anxiety on HADS, and that patient calls the AVR 24/7 phone to elaborate on and get advice for a case he felt anxious about after sAVR. This would validate information that stems from the instrument.

Prevention of missing data to increase the representativeness of the sample in this trial is related to both designing and conducting the trial [32]. In designing the intervention, former patients and interdisciplinary specialists in the cardiac field revealed the themes for the intervention manual and 24/7 follow-up after discharge, in accordance with evidence-based literature. Moreover, the intervention is flexible, as it is based on when the patients need support. The patients in the control group receive information at discharge about group allocation and the importance of comparing the intervention and the control group in order to offer future sAVR patients a solid follow-up based on patients’ needs. When conducting the study, the participants’ burden and inconvenience of data collection is limited to only two questionnaires with a few items, to avoid missing values and drop-out. The project coordinator is dedicated to follow up the participants and the expert nurses responsible for the 24/7 intervention to limit missing data in the conduct of the study. It is time-consuming to carry out a 24/7 phone support service, and it requires expert healthcare professionals to be deeply and continuously motivated to seriously carry out the study. This is an ongoing challenge, and it is necessary to safeguard the strength of the study. The intervention is bolstered by experiencing and discussing patient cases and by the teaching of relevant themes during the intervention period. Moreover, assessment of the intervention’s cost utility will provide valuable information for the healthcare system to develop ways to improve the transition of patient care to reduce readmissions [35]. Furthermore, knowledge from this study may add valuable information to optimize healthcare for future comparison to the emerging transcatheter aortic valve implantation (TAVI) patient population.

Insight into an individual patient’s pathway through the readmission process is made possible for the first time by patients’ agreeing to allow researchers to gain access to register data. A normal pathway for a patient undergoing sAVR is to be transferred from an index, specialised hospital to a non-index hospital with a lower level of care at the fourth day after surgery. Fragmented care, which can occur when patients are transferred between hospitals at different levels in healthcare systems, increases the risk of mortality [5] and is a present challenge for patients and the healthcare system. This study is limited in that it includes patients only at one university hospital with two departments.

Before surgery, the patients are informed about the expected increase in health status after surgery. Adding the QALY analysis takes into account both the quantity and quality of life generated by healthcare intervention and may add valuable preoperative information for future patients undergoing sAVR.

The lack of masking in this study related to patients may have a potential influence on outcomes [24]. Patients are informed about group allocation 1–2 days before discharge from the University Hospital. If a patient from the control group and one from the intervention group by coincidence are in the same room, the project nurse has organised separate information about further follow-up in the study. The patient in the intervention group is encouraged not to share information about the intervention. The general information of the ongoing AVRre study at the Department might influence the patient in the control group and the caregivers, e.g. to offer more information than usual care and possibly threaten internal validity (the Hawthorne effect).

In conclusion, the knowledge gained from this study will provide valuable insights for adjusting aspects of the healthcare system now to improve care for patients undergoing sAVR and will inform future studies on sAVR. The 24/7 phone-support manual has the potential to be modified and adopted for use by other surgical populations with high readmission rates.

### Trial status at the time of initial manuscript submission

Recruitment for this trial is ongoing.

## Additional files

Additional file 1: SPIRIT 2013 checklist: recommended items to address in a clinical trial protocol. (DOC 122 kb)

Additional file 2: Supporting material for the AVRre study. (DOCX 82 kb)

Additional file 3: Written informed consent form. (DOC 49 kb)

## Abbreviations

DRG: Diagnosis-related group
EQ-5D: EuroQol-5 dimensions
HADS: Hospital Anxiety and Depression Scale
HRQOL: Health-related quality of life
QALY: Quality-adjusted life year (QALY)
sAVR: Surgical aortic valve replacement
