# Supplementary material for: Determining the impact of 24/7 phone support on hospital readmissions after aortic valve replacement surgery (the AVRre study): study protocol for a randomised controlled trial
Source: Trials. 2017 May 30;18:246. doi: 10.1186/s13063-017-1971-y (PMC6389149; doi:10.1186/s13063-017-1971-y)
Supplement: Supplementary file 2 — Supporting material for the AVRre study. (DOCX 82 kb) [file 13063_2017_1971_MOESM2_ESM.docx]

**SUPPORTING MATERIAL**

**Brief description of the development of the 24/7-phone support manual for the AVRre study, and an example section**

We first sought guidance from previous research and healthcare professional experiences to guide development of the 24/7 phone-support manual. Evidence-based literature searches, search of qualitative, research interviews with former heart surgery patients (2+2+1) and interviews with interdisciplinary specialists in the cardiac field revealed 11 consensus themes that were relevant. These themes were: 1. Dyspnoea (example table 1); 2. Heart rhythm; 3. Pain; 4. Prescribed medication**;** 5. Infection (Hygiene); 6. Psychological (anxiety, depression, cognitive)**;** 7. Activity (daily)**;** 8. Nutrition/Lifestyle (diet/alcohol/smoke)**;** 9. Social network**;** 10. Sexuality; and 11. Others. The overarching goal of the manual is to help patients understand and self-manage non-urgent symptoms at home. Based on symptoms the patients present, apparent clinical signs described, and questions they ask when calling the intervention phone line, the project nurses use advice and recommended action patterns (like an algorithm), organized according to a universal model of color-coded severity, that being red (acute), yellow (urgent) and green (non-urgent) response.

Table 1 Dyspnoea example from 24/7-phone support manual

|  | **Criteria** | **Symptoms/Clinical signs/Questions** | **Advice** |
| --- | --- | --- | --- |
| ACUTE | A.1.01 | Intense; by speech (breathlessness at speech -> tamponade?), or fast breathing and high pulse, pain (epigastric), dizzy, pale, sick, loss of appetite, extreme anxiety, restlessness, deteriorating condition (late tamponade?) | 1,2 |
|  | A.1.02 | Intense; intense retrosternal pain (secondary cardiac event like a myocardial infarction?) | 1,2 |
|  | A.1.03 | Intense; fever >38.5° C with chills, cough and sputum (pneumonia high fever ->endocarditis?) | 1,2 |
|  | A.1.04 | Intense; Next of kin is calling. Patient unable to talk? (Thrombus with apoplexy? Or other serious illness?) | 1,2 |
|  | | | |
| URGENT | H.1.01 | Moderate; chest pain and deteriorating condition | 3-6 |
|  | H.1.02 | Moderate; chest pain when moving, but is active | 3-5, 8 |
|  | H.1.03 | Moderate; fever <38.5°C, coughing and not able to walk up stairs | 3-7 |
|  | H.1.04 | Moderate; wound infection? (red, swollen, pain, secretions?) | 3-5 |
|  | H.1.05 | Moderate; extremely anxious about their health | 3-5, 13 |
|  | | | |
| NON-URGENT | V.1.01 | Lighter; by activity, but no pain | 6,11 |
|  | V.1.02 | Lighter; some wound pain and some feeling of reduced general condition | 6,8,9 |
|  | V.1.03 | Lighter; sleeping poorly and experiences fatigue often | 6,7,9,14 |
|  | V.1.04 | Lighter; some anxiety for disease associated to the surgery | 6,10,12,13 |
|  | V.1.05 | Lighter; insecure related to physical activity/training | 6,8,11 |
|  | V.1.06 | Others |  |

The intervention is strengthened by the experience and discussion of patient cases and by teaching of relevant themes during the intervention period.
