# Supplementary material for: Determining the impact of 24/7 phone support on hospital readmissions after aortic valve replacement surgery (the AVRre study): study protocol for a randomised controlled trial
Source: Trials. 2017 May 30;18:246. doi: 10.1186/s13063-017-1971-y (PMC6389149; doi:10.1186/s13063-017-1971-y)
Supplement: Supplementary file 3 — Written informed consent form. (DOC 49 kb) [file 13063_2017_1971_MOESM3_ESM.doc]

**Request for participation in a research project**

***Around-the-clock phone support the first month***

***after surgical aortic valve replacement surgery***

**Background and aim**

This is a question to you about participating in a research study to test a new offer of around-the-clock phone support the first month after surgical aortic valve replacement surgery. The aim of the study is to contribute to patients feeling of security, and to get advices and counselling the first month after discharge from hospital. Research have shown that patients’ needs more follow up and support the first month after discharge. The study will survey if this follow up will result in reduced numbers of readmissions and reduced number of general practitioner consultations. The study will be performed at Department of Cardiothoracic Surgery, Oslo University Hospital (OUH), Ullevål and Rikshospitalet. Oslo University Hospital is responsible for the study. The results from the study will be used to improve the future offer to surgical aortic valve replacement surgery patients after discharge.

**What contains the study?**

All participants in the study will be asked to fill in the received questionnaires to survey symptoms on anxiety and depression and quality of life. You will be asked to fill in a scheme about eventually other diseases. It is estimated to take about 10 minutes to fill in the schemes. To achieve knowledge about your condition over time, you will be asked to fill in the same questionnaires received by postal mail in pre stamped envelopes at 1,3 6, and 12 months after discharge. In addition, we ask for your permission to collect data on readmissions to hospital and general practitioner consultations. We will get access to this data due to application to the Norwegian Directorate of Health. All patients hospitalised for surgical aortic valve replacement surgery will be invited to participate in the study.

All patients consenting to participate in the study will be randomly divided into two groups. In that way we can compare if the phone support intervention has an effect.

If you are divided into **group 1** the following will happen:

- You will answer questionnaires 5 times: before surgery, 1,3, 6 and 12 months after discharge.
- You can call the intervention phone (separate written patient leaflet later) around-the-clock the first month after discharge. An expert nurse will give you advice and eventually consult medical doctor specialists at the hospital.
- You will be called 2 and 9 days after discharge to ensure that you are doing well and eventually get answer to questions. You will receive an SMS a day ahead, to eventually be able to change to a day that is more suitable for you.

If you are divided into **group 2** the following will happen:

- You will answer questionnaires 5 times: before surgery, 1,3, 6 and 12 months after discharge.

## Possible advantages and disadvantages

The study involves no costs, and there are no risks associated with the study. You may experience it a little tiresome or stressful to answer the questions.

## What happens with the information about you?

The registered information about you will be uses as described in the aim of the study. All information will be treated without name and identity number or other direct recognition information. A code connects you to your information through a name list. The name list and codes will be deleted when the study is completed. We plan to complete the study in 2020. Only authorized health care professionals participating in the study will have access to the name list and that can get back to you. As part of the study we want to collect information about yours diagnose, treatment, eventually other diseases and medication from the medical record at the hospital. You are entitled to access to the information being recorded. Answers to questionnaires will be analysed with data of readmissions and general practitioner consultations, which we are given access to by the Norwegian Directorate of Health. When the results of the study are published it will not be possible to identify you in the results.

The study is recommended by Regional Ethical Committee, The Data Protection Officer at Oslo University Hospital and the management at Department of Cardiothoracic Surgery, Oslo University Hospital (OUH).

**Volunteer participation**

It is voluntary to participate in the study. You can withdraw your consent at any time without any explanation. This will not affect your further treatment. If you decide to participate, you will sign the declaration of consent as follows. If you consent to participate, you can withdraw your consent without any consequences for your treatment. You can require that existing data be deleted. Data that has been analysed and published can not be withdrawn.

If you want to withdraw or have questions about the study, you can contact project leader Irene Lie on e-mail: [irene.lie@ous-hf.no](mailto:irene.lie@ous-hf.no), cell phone 99026729.

Best regards

Irene Lie

# Written informed consent to participate in the study

I am willing to participate in the study

-----------------------------------------------------------------------------------------------------------------

(Signature by study participant, date)

I can be contacted at the following phone: ______________ Alt. Phone:_______________

Email adr:__________________________________________________________________

I confirm to have given information about the study

-----------------------------------------------------------------------------------------------------------------

(Signature, role in the study, date)
